# C188-9, a specific inhibitor of STAT3 signaling, prevents thermal burn-induced skeletal muscle wasting in mice

**DOI:** 10.3389/fphar.2022.1031906

**Published:** 2022-12-16

**Authors:** Yuko Ono, Masafumi Saito, Kazuho Sakamoto, Yuko Maejima, Shingen Misaka, Kenju Shimomura, Nobuto Nakanishi, Shigeaki Inoue, Joji Kotani

**Affiliations:** ^1^ Department of Disaster and Emergency Medicine, Graduate School of Medicine, Kobe University, Kobe, Japan; ^2^ Department of Bioregulation and Pharmacological Medicine, School of Medicine, Fukushima Medical University, Fukushima, Japan; ^3^ Department of Bio-Informational Pharmacology, School of Pharmaceutical Sciences, University of Shizuoka, Shizuoka, Japan

**Keywords:** humoral factor, hyper catabolism, interleukin-6, pharmacological intervention, skeletal muscle atrophy, systemic inflammatory response syndrome, ubiquitin proteasome pathway

## Abstract

Burn injury is the leading cause of death and disability worldwide and places a tremendous economic burden on society. Systemic inflammatory responses induced by thermal burn injury can cause muscle wasting, a severe involuntary loss of skeletal muscle that adversely affects the survival and functional outcomes of these patients. Currently, no pharmacological interventions are available for the treatment of thermal burn-induced skeletal muscle wasting. Elevated levels of inflammatory cytokines, such as interleukin-6 (IL-6), are important hallmarks of severe burn injury. The levels of signal transducer and activator of transcription 3 (STAT3)—a downstream component of IL-6 inflammatory signaling—are elevated with muscle wasting in various pro-catabolic conditions, and STAT3 has been implicated in the regulation of skeletal muscle atrophy. Here, we tested the effects of the STAT3-specific signaling inhibitor C188-9 on thermal burn injury-induced skeletal muscle wasting *in vivo* and on C2C12 myotube atrophy *in vitro* after the administration of plasma from burn model mice. In mice, thermal burn injury severity dependently increased IL-6 in the plasma and tibialis anterior muscles and activated the STAT3 (increased ratio of phospho-STAT3/STAT3) and ubiquitin-proteasome proteolytic pathways (increased Atrogin-1/MAFbx and MuRF1). These effects resulted in skeletal muscle atrophy and reduced grip strength. In murine C2C12 myotubes, plasma from burn mice activated the same inflammatory and proteolytic pathways, leading to myotube atrophy. In mice with burn injury, the intraperitoneal injection of C188-9 (50 mg/kg) reduced activation of the STAT3 and ubiquitin-proteasome proteolytic pathways, reversed skeletal muscle atrophy, and increased grip strength. Similarly, pretreatment of murine C2C12 myotubes with C188-9 (10 µM) reduced activation of the same inflammatory and proteolytic pathways, and ameliorated myotube atrophy induced by plasma taken from burn model mice. Collectively, these results indicate that pharmacological inhibition of STAT3 signaling may be a novel therapeutic strategy for thermal burn-induced skeletal muscle wasting.

## Introduction

Burn injury, the fourth most common type of trauma, is a leading cause of death and disability worldwide (World Health Organization, 2014). Recent estimates indicate that the annual global incidence of severe burn injury is nine million cases, resulting in approximately 120,000 deaths annually (James et al., 2020). Burns are one of the top 10 leading causes of death among young people aged 0–29 years (World Health Organization. 2014) and are ranked seventh in the top 10 causes of disability-adjusted life years for adults aged 15–44 years (Ribeiro et al., 2008; Chandran et al., 2010). Burns are therefore a major healthcare concern worldwide that warrants increasing public awareness.

Persistent systemic inflammatory responses, hypermetabolism, and skeletal muscle loss are distinct features of severe burn injuries (Merritt et al., 2012; Shelhamer et al., 2015; Kashiwagi et al., 2017). Among various proinflammatory cytokines, interleukin (IL)-6 is an especially important hallmark of severe burn injuries because plasma IL-6 concentrations were correlated with burn severity and increased mortality (Pileri et al., 2008; Lantos et al., 2010; Shelhamer et al., 2015). In addition to the burn injuries, increased levels of circulatory IL-6 are present in other medical conditions, including sepsis (Zanders et al., 2022), COVID-19 (Kang et al., 2020; Ali and Kunugi, 2021; Mittal et al., 2021; Seixas et al., 2022; Soares et al., 2022), diabetes mellitus (Perry et al., 2016), cancer (Rupert et al., 2021), chronic obstructive pulmonary disease (Lin et al., 2021), myocardial failure (Janssen et al., 2005), and end-stage kidney disease (Zhang et al., 2013). The severe involuntary loss of skeletal muscle, termed muscle wasting, is observed in all these conditions, suggesting a potential role for IL-6 in their development (Muñoz-Cánoves et al., 2013; Guadagnin et al., 2018). We recently extended these observations by demonstrating that endotoxin increased plasma IL-6 protein concentrations and skeletal muscle IL-6 mRNA expression, and induced skeletal muscle atrophy in mice (Ono et al., 2020). Skeletal muscle wasting adversely affects both the survival and functional outcomes of patients, and increases an economic burden. For example, skeletal muscle weakness was related to prolonged length of mechanical ventilation and intensive care unit stay; increased mortality; and long-term physical disabilities among critically ill patients (Ali et al., 2008; Hermans et al., 2014; Wang et al., 2020). Thus, there is an imperative need to advance therapeutic interventions for skeletal muscle wasting associated with systemic inflammation.

The Janus kinase (JAK)/signal transducer and activator of transcription 3 (STAT3) signaling pathway has a crucial role in muscle wasting induced by IL-6 (Muñoz-Cánoves et al., 2013; Guadagnin et al., 2018). Previous studies showed that the phosphorylation of STAT3 is associated with the development of skeletal muscle atrophy in various conditions, including burns (Corrick et al., 2015; Kashiwagi, et al., 2017; Pin et al., 2019), sepsis (Zanders et al., 2022), cancer (Bonetto, et al., 2011; Silva et al., 2015), degenerative muscle disease (Wada et al., 2017; Liang et al., 2018), immobilization (Huang, et al., 2020; Hirata et al., 2022), and chronic kidney diseases (Zhang et al., 2013). Previous studies reported that the IL-6/STAT3 pathway increased the expressions of skeletal muscle ubiquitin E3 ligases MuRF1 and Atrogin-1/MAFbx, which are major cellular proteolytic systems responsible for skeletal muscle atrophy (Bonetto et al., 2012; Silva et al., 2015; Sala and Sacco, 2016; Guadagnin et al., 2018). Consistent with these experimental findings, increased ubiquitin-proteasome activity was found in muscle biopsies from patients with severe burns (Merritt et al., 2012; Merritt et al., 2013). These observations collectively support the idea that inhibiting STAT3 signaling might abrogate skeletal muscle wasting induced by factors downstream of the IL-6 inflammation pathway.

C188-9, which is a specific inhibitor of STAT3 signaling, inhibits the phosphorous peptide binding site within STAT3 Src homology two domains with high affinity (Xu, et al., 2009; Redell, et al., 2011; Bharadwaj, et al., 2016). Previous studies showed that C188-9 prevented chronic kidney failure-induced skeletal muscle atrophy (Zhang et al., 2013), cancer-induced skeletal muscle atrophy (Silva et al., 2015), and denervation-induced skeletal muscle atrophy (Huang, et al., 2020) by suppressing activation of proteolytic pathways, including ubiquitin-proteasome degradation in skeletal muscle. Importantly, STAT3 inhibitors were used in a clinical trial of patients with cancer and were generally well tolerated (Oh et al., 2015; Wong et al., 2015). However, no reports have yet focused on the impact of pharmacological STAT3 inhibition on skeletal muscle wasting during thermal burn insults.

In this study, we evaluated the effect of STAT3 inhibition by C188-9 on thermal burn-induced skeletal muscle wasting in mice. Our *in vivo* and *in vitro* experimental results show a beneficial effect of C188-9 on thermal burn-induced skeletal muscle wasting.

## Results

### Thermal burn injury induces skeletal muscle atrophy and weakness in mice in a severity-dependent manner

**FIGURE 1:**
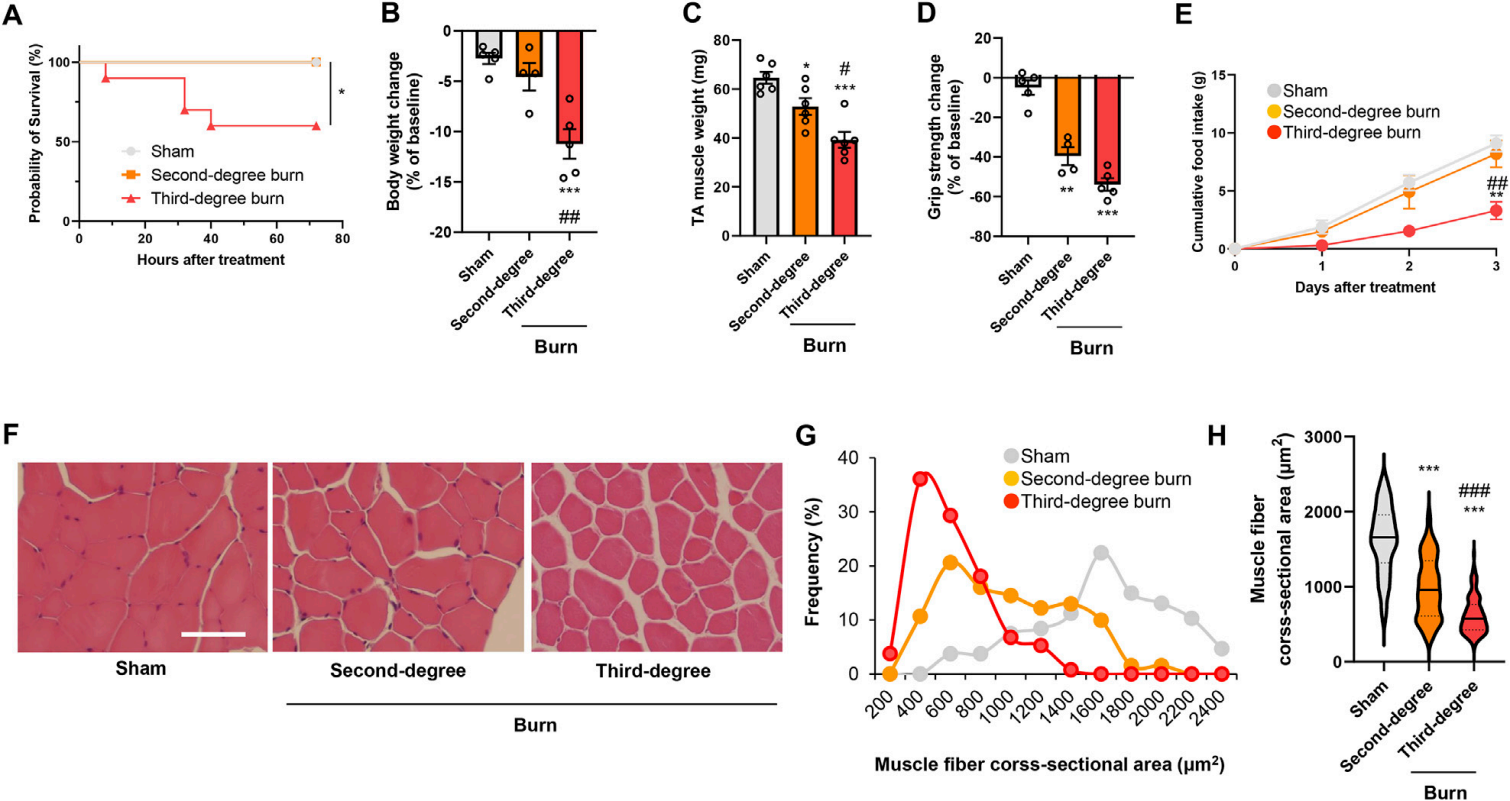
Thermal burn injury induces muscle atrophy and weakness in mice by a severity dependent mechanism. Second-degree, third-degree, or sham burns were administered to wild-type C57BL/6 mice (12–16-week-old male mice). **(A)** Cumulative survival after burn or sham burn injury. N = 5–10/group. *p < 0.05 by log rank test. After 3 days of burn or sham-burn injury, mice body weight **(B)**, TA muscle weight **(C)**, and grip strength **(D)** were assessed. Food intake **(E)** was measured every 24 h up to 72 h. N = 4–6/group. Representative images of H&E-stained TA muscle sections **(F)** and quantification of the distribution **(G)** and violin plot **(H)** of cross-sectional areas of TA muscle fibers 3 days after thermal burn injury. The continuous lines and dotted lines within the violin plot indicate the median and quartiles, respectively. The cross-sectional area of TA muscle fibers was measured. Scale bar, 50 µm. N = 106–133/group. For all panels, data are presented as the mean ± s.e.m. ***p < 0.001, **p < 0.01, *p < 0.05 vs. sham control group, ###*p* < 0.001, ##*p* < 0.01, #*p* < 0.05 vs. second-degree burn group by one-way ANOVA followed by Tukey’s honest significant difference test.

Thermal burn injury is associated with skeletal muscle wasting (Corrick et al., 2015; Kashiwagi, et al., 2017; Pin et al., 2019), but how the burn severity affects skeletal muscle atrophy has not been fully determined. We therefore developed two different mouse models of thermal burn injuries (second-degree burn and third-degree burn) as described in the Methods. No mortality was observed for 72 h following a sham-burn or second-degree burn injury, whereas the mortality rate was approximately 40% after third-degree burn injuries (Figure 1A). In surviving mice, a thermal burn injury caused body weight loss (Figure 1B), tibialis anterior (TA) muscle loss (Figure 1C), and grip strength loss (Figure 1D) in a severity-dependent manner. At the end of the experiment, mice with sham or second-degree burns ate an adequate amount of food, whereas mice with third-degree burn injuries were lethargic and had lost their appetite (Figure 1E). The histological analysis of TA muscle sections showed that thermal burn severity dependently induced skeletal muscle atrophy demonstrated by the shrinkage of muscle fibers and increased the interstitial space (Figures 1F–H). Collectively, these results suggest that thermal burn injury induces skeletal muscle wasting in mice in a severity-dependent manner.

### Thermal burn injury activates STAT3 and ubiquitin-proteasome pathways and reduces PI3K/Akt pathway activation in mice in a severity-dependent manner

**FIGURE 2:**
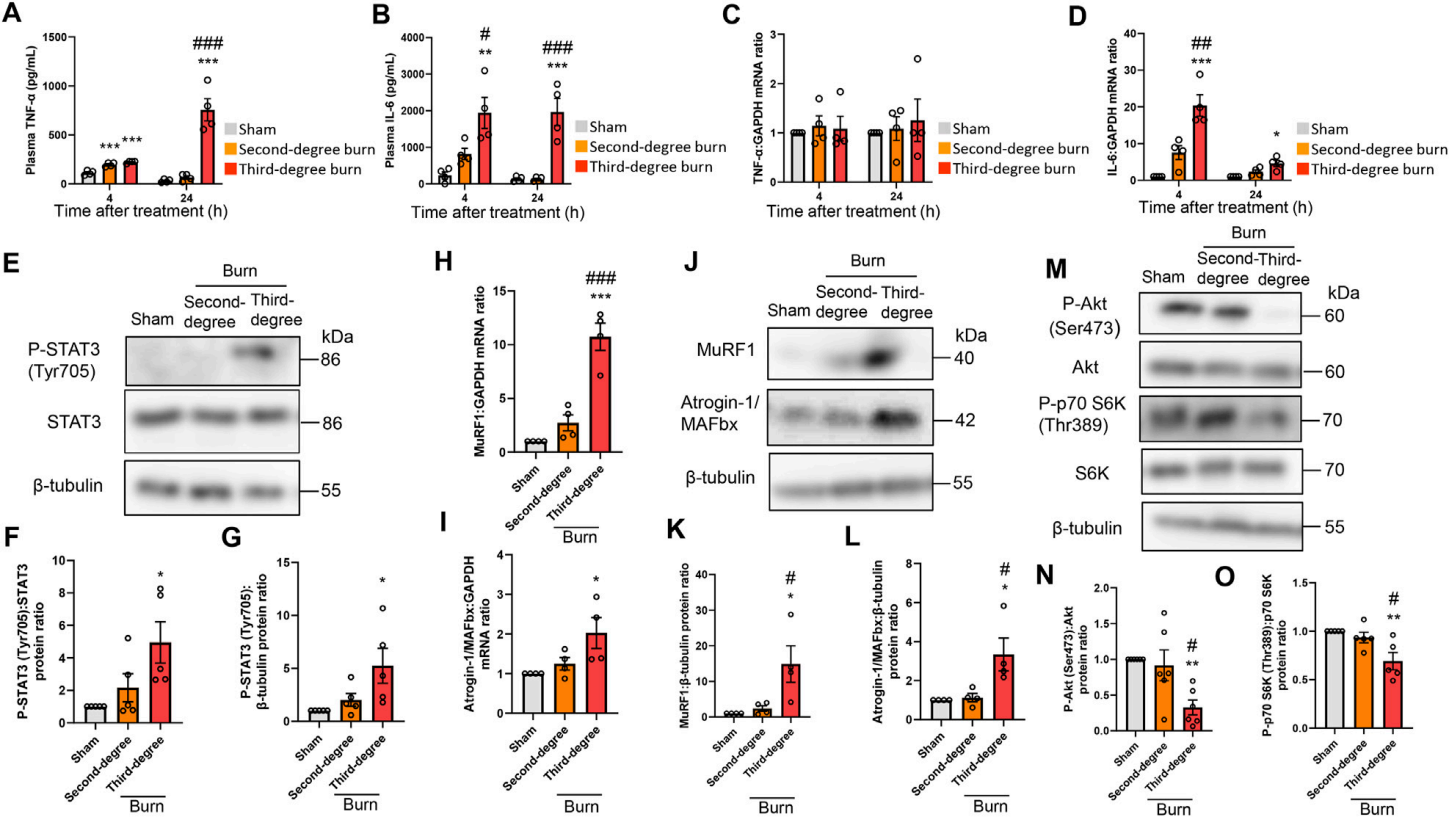
Thermal burn injury activates STAT3 and muscle proteolytic pathways, and reduces muscle anabolic pathways in mice by a severity dependent mechanism. Second-degree, third-degree, or sham burns were administered to wild-type C57BL/6 mice (12–16-week-old male mice) and 4 and 24 h after burn or sham-burn injury, plasma samples were prepared and TNF-α **(A)** and IL-6 **(B)** concentrations were measured by ELISA. N = 4/group. qRT-PCR analysis of TNF-α **(C)** and IL-6 **(D)** mRNAs in TA muscles 4 and 24 h after burn or sham-burn injury. Data were normalized to GAPDH mRNA levels and are shown as fold increase over the sham controls. N = 4/group. Western blot analysis **(E)** and quantification of P-STAT3 **(F,G)** in TA muscles 24 h after burn or sham-burn. Data were normalized to STAT3 protein levels **(F)** or β-tubulin protein levels **(G)** and the ratio in sham-control mice was set as 1. N = 5/group. qRT-PCR analysis of MuRF1 **(H)** and Atrogin-1/MAFbx **(I)** mRNAs in TA muscles 24 h after burn or sham-burn injury. Data were normalized to GAPDH mRNA levels and are shown as fold increase over the sham controls. N = 4/group. Western blot analysis **(J)** and quantification of MuRF1 **(K)** and Atrogin-1/MAFbx **(L)** expressions in TA muscles 24 h after burn or sham-burn treatment. Data were normalized to β-tubulin protein levels and the ratio in sham-control mice was set as 1. N = 4/group. Western blot analysis **(M)** and quantification of P-Akt **(N)** and P-p70 S6K **(O)** expressions in TA muscles 24 h after burn or sham-burn treatment. Data were normalized to Akt and p70 S6K protein levels, respectively, and the ratio in sham-control mice was set as 1. N = 5–6/group. For all panels, data are presented as the mean ± s.e.m. ***p < 0.001, **p < 0.01, *p < 0.05 vs. sham control group, ###*p* < 0.001, ##*p* < 0.01, #*p* < 0.05 vs. second-degree burn group by one-way ANOVA followed by Tukey’s honest significant difference test.

Thermal burn increased circulating proinflammatory cytokines, including tumor necrosis factor (TNF)-α and IL-6 (Merritt et al., 2012; Merritt et al., 2013), and these cytokines induced muscle proteolysis and atrophy (Haddad et al., 2005; Muñoz-Cánoves et al., 2013; Guadagnin et al., 2018). In humans, circulating proinflammatory cytokine levels, especially IL-6, were positively associated with burn severity and poor outcomes (Pileri et al., 2008; Lantos et al., 2010; Shelhamer et al., 2015). In accordance with these observations in human burn victims, thermal burn elevated plasma TNF-α and IL-6 concentrations in a severity-dependent manner (Figures 2A,B). Consistent with these findings in the plasma, burn insults increased the mRNA expression of IL-6, but not TNF-α, in the TA muscle of burn mice (Figures 2C,D). These results were also in line with a clinical study that examined skeletal muscle biopsies from human burn patients (Merritt et al., 2012; Merritt et al., 2013).

These findings prompted us to measure STAT3 pathway activation, which is a key downstream component of IL-6 inflammatory signaling, following burn injury (Haddad et al., 2005; Muñoz-Cánoves et al., 2013; Guadagnin et al., 2018). As shown in Figures 2E–G, thermal burn injury increased the phosphorylated (P)-STAT3/STAT3 protein ratio and P-STAT3/β-tubulin protein ratio by a severity-dependent mechanism in TA muscles from mice 24 h after burn insults. Then, we measured two muscle-enriched ubiquitin ligases, MuRF1 and Atrogin-1/MAFbx, which are key enzymes in the muscle proteolytic pathway (Bodine, et al., 2001; Glass, 2003). Because the STAT3 pathway increased the expressions of ubiquitin-proteasome pathway genes (Muñoz-Cánoves et al., 2013; Guadagnin et al., 2018), we speculated that the ubiquitin ligase mRNA expressions would be increased after thermal burn injury. As predicted, we found that thermal burn injury significantly increased the MuRF1 and Atrogin-1/MAFbx mRNA expressions of TA muscles in mice by a severity dependent mechanism (Figures 2H,I). To extend these observations at the protein level, we measured MuRF1 and Atrogin-1/MAFbx protein expressions. In agreement with the findings in Figures 2H,I, western blot analysis showed that the expressions of both ubiquitin ligase proteins were upregulated in TA muscles 24 h after burn insult, by a severity dependent mechanism (Figures 2J–L).

In addition to the ubiquitin-proteasome system, apoptosis has an important role in muscle protein degradation and was implicated in various catabolic conditions associated with muscle atrophy (Du et al., 2004; Supinski and Callahan, 2006). To evaluate the effect of thermal burn injury on apoptosis signals in our model, we examined the expression of cleaved caspase-3, an indicator of apoptosis signaling 24 h after burn injuries. We speculated that the apoptosis signal might not change in our model during the predetermined time course because caspase-3 is a downstream component of TNF-α signaling (Wajant, et al., 2003), and TNF-α mRNA expression in skeletal muscle remained unchanged 24 h after burn insults. As predicted, western blot analysis showed cleaved caspase-3 protein levels, the active form of caspase-3, were not increased in TA muscle 24 h after burn injury (Supplementary Figures S1A,B in the Supplementary Material). Bcl-2- and Bax-mediated pro-apoptotic signaling also plays a crucial role in skeletal muscle atrophy associated with cancer (Alves et al., 2019; Miao et al., 2021), denervation (Siu and Alway, 2005), and pressure-induced injury (Tam et al., 2018). Therefore, we next evaluated expression of the anti-apoptotic protein Bcl-2 and pro-apoptotic protein Bax, and the Bax/Bcl-2 protein ratio in our model. Consistent with the findings of our cleaved caspase-3 analysis, none of these proapoptotic parameters were altered in TA muscle 24 h after burn injury (Supplementary Figure S1A and Supplementary Figures S1C–E in the Supplementary Material).

Impaired cell growth signals are another crucial determinant of skeletal muscle atrophy (Cohen et al., 2015; Friedrich et al., 2015; Sartori et al., 2021). Among the growth signals that control muscle size, the PI3K/Akt pathway especially plays an important role in blocking protein degradation (Schiaffino and Mammucari, 2011; Fanzani et al., 2012). Akt activates S6 kinase (S6K) *via* mammalian target of rapamycin, which leads to an increase in protein synthesis (Schiaffino and Mammucari, 2011; Fanzani et al., 2012). We next investigated the effect of burn severity on the PI3K/Akt pathway in the skeletal muscle of mice. In agreement with previous findings (Lang et al., 2004; Duan et al., 2009; Corrick et al., 2015; Pin et al., 2019), the phosphorylation of p70 S6K (Thr389) and Akt (Ser473) were decreased in TA muscles 24 h after burn injury, which was severity dependent (Figures 2M–O). Collectively, these results suggested that thermal burn injury activated STAT3 and muscle proteolytic pathways while reducing muscle anabolic pathways by a severity dependent mechanism.

### Plasma from burned mice directly induces protein catabolism that promotes C2C12 myotube atrophy *in vitro*

**FIGURE 3:**
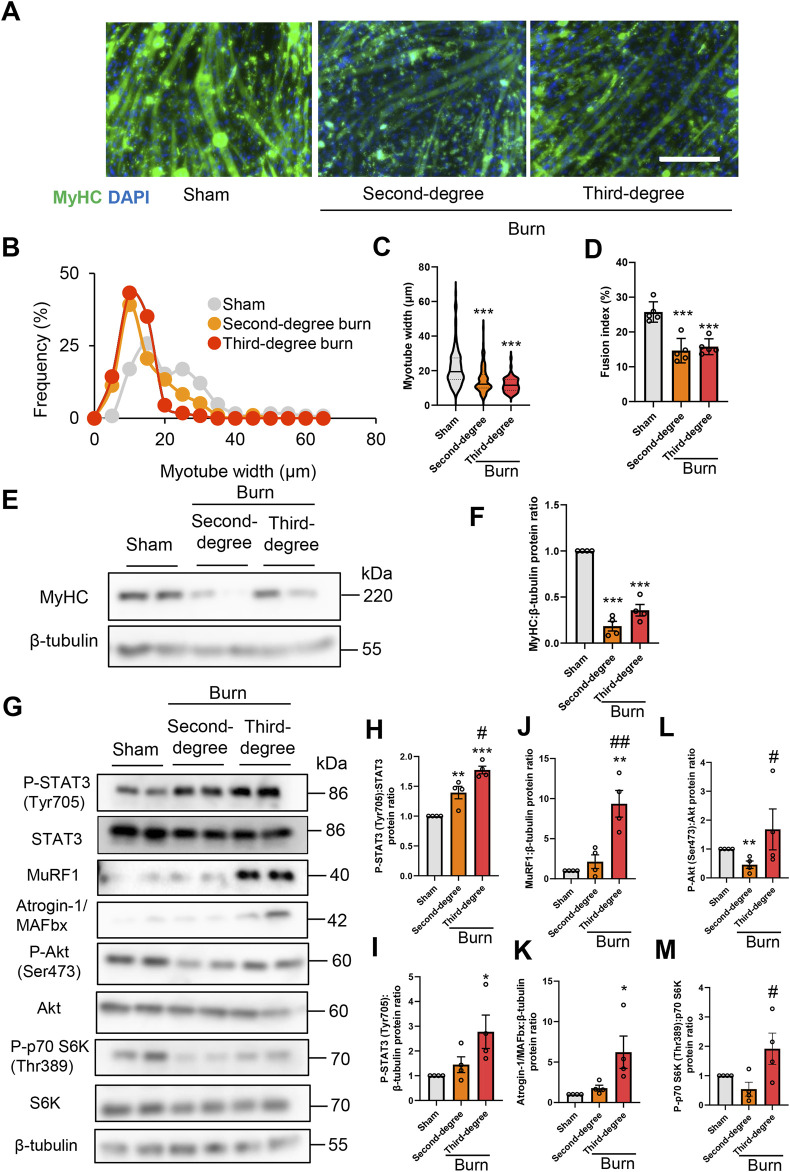
Plasma from burned mice induces the atrophy of C2C12 myotubes. C2C12 myotubes were incubated with plasma collected from burn or sham-burn mice (5% vol/vol) for 24 or 48 h. Representative immunofluorescence staining of MyHC in C2C12 myotubes **(A)** treated with plasma from burn or sham-burn mice for 48 h. Scale bar = 100 µm. Distribution of myotube widths **(B)**, violin plot of myotube width **(C)**, and fusion index **(D)**. N = 97–124/group. The continuous lines and dotted lines within the violin plot indicate the median and quartiles, respectively. The fusion index was calculated from five randomly selected fields. Western blot analysis **(E)** and quantification **(F)** of MyHC expression in C2C12 myotubes treated for 48 h with plasma from burn or sham-burn mice. Data in **(F)** were normalized to β-tubulin protein levels, and the ratio in sham control cells was set as 1. N = 4/group. Western blot analysis **(G)** and quantification of P-STAT3 **(H,I)**, MuRF1 **(J)**, Atrogin-I/MAFbx **(K)**, P-Akt **(L)**, and P-p70 S6K **(M)** in C2C12 myotubes treated with plasma from burn or sham-burn mice for 24 h. Data were normalized to STAT3, β-tubulin, Akt, and p70 S6K protein levels, respectively, and the ratio in sham control cells was set as 1. N = 4/group. For all panels, data are presented as the mean ± s.e.m. ***p < 0.001, **p < 0.01, *p < 0.05 vs. cells treated with plasma from mice with sham burn; ##*p* < 0.01 #*p* < 0.05vs. cells treated with plasma from mice with second-degree burns. P-values were derived from one-way ANOVA followed by Tukey’s honest significant difference test or Kruskal-Wallis test followed by Dunn’s *post hoc* tests with Bonferroni correction.

To examine the direct effect of humoral factors related to burns on skeletal muscle, C2C12 myotubes were cultured for 24 or 48 h in the presence of plasma collected from mice with sham burns, second-degree burns, or third-degree burns that comprised 20% of their total body surface area as described in the Methods. Consistent with *in vivo* findings in mice, the immunofluorescence staining of myotubes with a myosin heavy chain (MyHC) specific antibody revealed that exposure to plasma from burn mice reduced the diameter of myotubes (Figures 3A–C). To quantify the morphological changes further, the fusion index (see the Methods) was calculated. As shown in Figure 3D, the fusion index was significantly lower in myotubes administered plasma from burn mice. These results collectively indicated that humoral factors from burn mice directly induced myotube atrophy in C2C12 cells.

We also used western blotting to confirm that plasma from burn mice suppressed the expression of the differentiation marker MyHC, but not by a severity dependent mechanism (Figures 3E,F). In accordance with the responses of mice *in vivo*, the active form of STAT3 and two ubiquitin ligase proteins were induced by plasma from burn mice by a severity dependent mechanism (Figures 3G–K). These results collectively suggested that humoral factors from burn mice directly induced STAT3 pathway activation, protein catabolism, and C2C12 myotube atrophy *in vitro*. In contrast, plasma from mice with second-degree burn injuries had impaired phosphorylation of Akt and S6K, whereas plasma from mice with third-degree burn injuries activated this pathway rather than inhibited it (Figure 3G, Figures 3L,M). Therefore, there might be an offset between catabolic and anabolic signals in C2C12 myotubes administered plasma from mice with third-degree burn injuries. This may partially explain why plasma from burn-injured mice decreased MyHC protein levels by a mechanism that was not severity dependent.

As shown in Figure 2B, plasma IL-6 concentrations were much higher in mice with third-degree burn injuries than those with second-degree burns or sham burns 24 h after treatment. A previous study showed that the JAK/STAT3 signaling pathway was associated with the promotion of myoblast proliferation and differentiation, but strongly activated catabolic signals, leading to myotube atrophy *in vitro* (Corrick et al., 2015; Quintana et al., 2015; Belizário et al., 2016; Yousuf et al., 2017). Therefore, murine recombinant IL-6 treatment might mimic the reaction of C2C12 myotubes treated with plasma from third-degree burn mice. To test this hypothesis, C2C12 myotubes were treated for 24 and 48 h with phosphate buffered saline (PBS), or murine recombinant IL-6 (100 ng/ml). This concentration of exogenous IL-6 was used based on relevant previous findings (Pelosi et al., 2014; Huang et al., 2020). As expected, the administration of recombinant IL-6 increased the P-STAT3/STAT3 protein ratio, activated catabolic and anabolic signals, and reduced the expression of MyHC protein (Supplementary Figure S2 in the Supplementary Materials).

### C188-9 prevents muscle wasting and weakness in mice with third-degree burns

**FIGURE 4:**
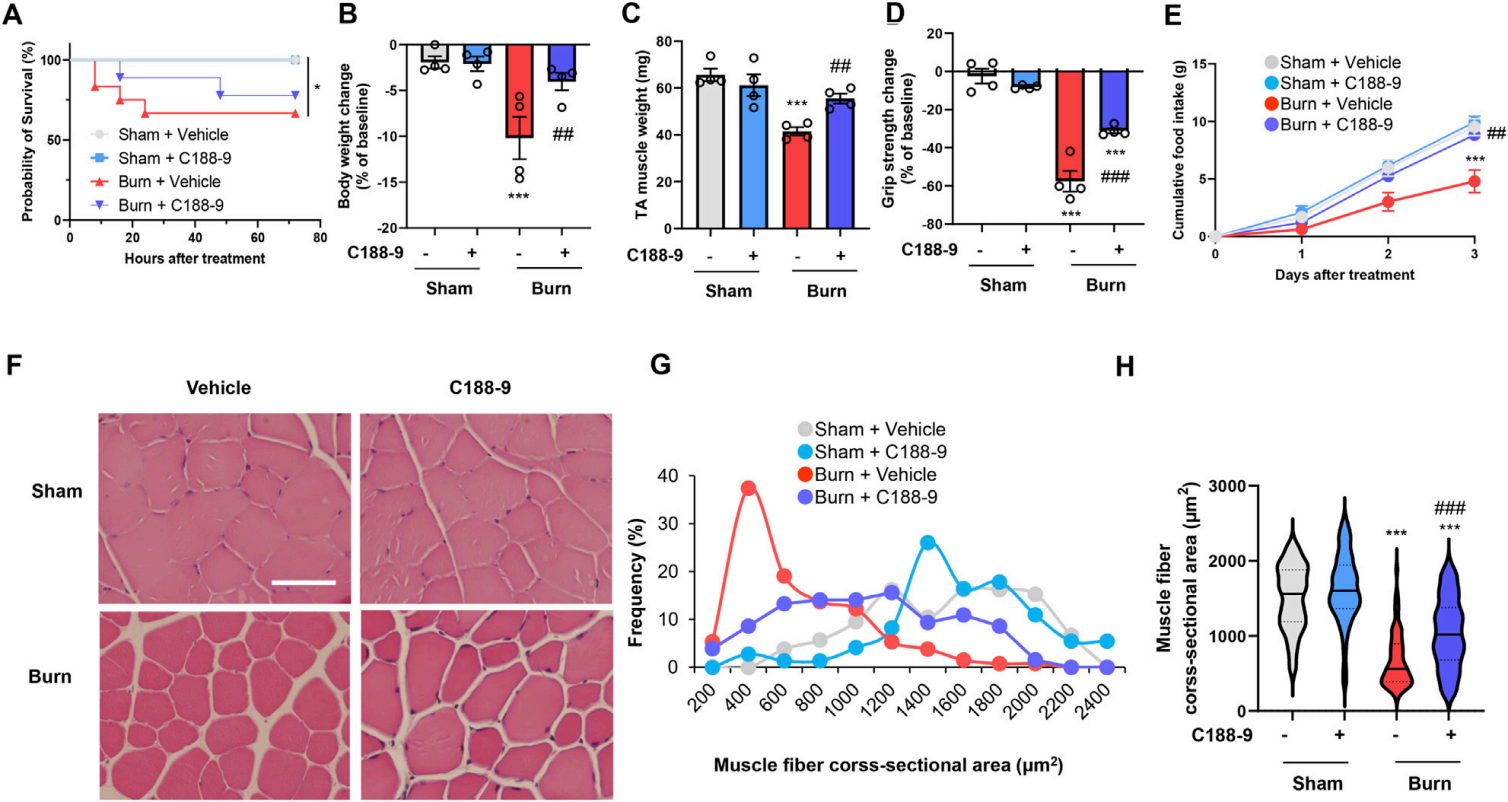
C188-9 reduces thermal burn-induced muscle atrophy and weakness in mice. Third-degree or sham burns were administered to wild-type C57BL/6 mice (12–16-week-old male mice) that were then ip injected with vehicle (5% wt/vol dextrose in distilled water containing 5% vol/vol DMSO) or C188-9 (50 mg/kg) 1 h later. The ip injection of C188-9 or vehicle was repeated every 24 h until sacrifice. **(A)** Cumulative survival after burn or sham-burn injury in mice given vehicle or C188-9. N = 9–11/group. *p < 0.05 by log rank test. After 3 days, mice were assessed for body weight **(B)**, TA muscle weight **(C)**, and grip strength **(D)**. Food intake **(E)** was measured every 24 h up to 72 h. N = 4–6/group. Representative images of H&E-stained TA muscle sections **(F)** and quantification of the distribution **(G)** and violin plot **(H)** of cross-sectional areas of TA muscle fibers 3 days after thermal burn injury. The continuous lines and dotted lines within the violin plot indicate the median and quartiles, respectively. Scale bar, 50 µm. N = 73–131/group. For all panels, data are presented as the mean ± s.e.m. ***p < 0.001 vs. sham injury with vehicle-treated group, ###*p* < 0.001, ##*p* < 0.01 vs. sham injury with vehicle-treated group by one-way ANOVA followed by Tukey’s honest significant difference test.

These findings suggested that we should examine the effect of STAT3 signaling inhibition on thermal burn-induced skeletal muscle wasting. Thus, male C57BL/6 mice aged 12–16 weeks were injected intraperitoneal (ip) with vehicle [5% wt/vol dextrose in distilled water containing 5% vol/vol dimethyl sulfoxide (DMSO)] or C188-9 (50 mg/kg) 1 h after third-degree burn injury or sham-burn injury. Sham-burn mice were injected ip with C188-9 or vehicle as corresponding controls. The ip injection of C188-9 or vehicle was repeated every 24 h until animals were sacrificed. The C188-9 doses and time course of administration were selected based on previous articles (Lewis et al., 2015; Bharadwaj et al., 2016; Gavino et al., 2016). Three days later, we assessed the body weight, muscle fiber size, and grip strength. Notably, whereas C188-9 administration did not affect acute phase mortality following burn injuries (Figure 4A), it significantly reversed thermal burn-induced body weight loss (Figure 4B), TA muscle loss (Figure 4C), and grip strength loss (Figure 4D) compared with vehicle-treated burn mice. Three days later, burn mice in the C188-9 injected group showed relatively normal behavior, eating a normal amount of food. In contrast, burn mice in the vehicle-injected group had lost their appetite (Figure 4E). The histological analysis of TA muscle sections showed that C188-9 ameliorated the thermal burn-induced shrinkage of muscle fibers (Figures 4F–H) compared with vehicle-treated mice. Collectively, these results indicate that the pharmacological inhibition of the STAT3 pathway restores thermal burn-induced skeletal muscle wasting histologically and functionally.

### C188-9 blocks skeletal muscle STAT3 and proteolytic signal activation in mice with third-degree burn injuries

**FIGURE 5:**
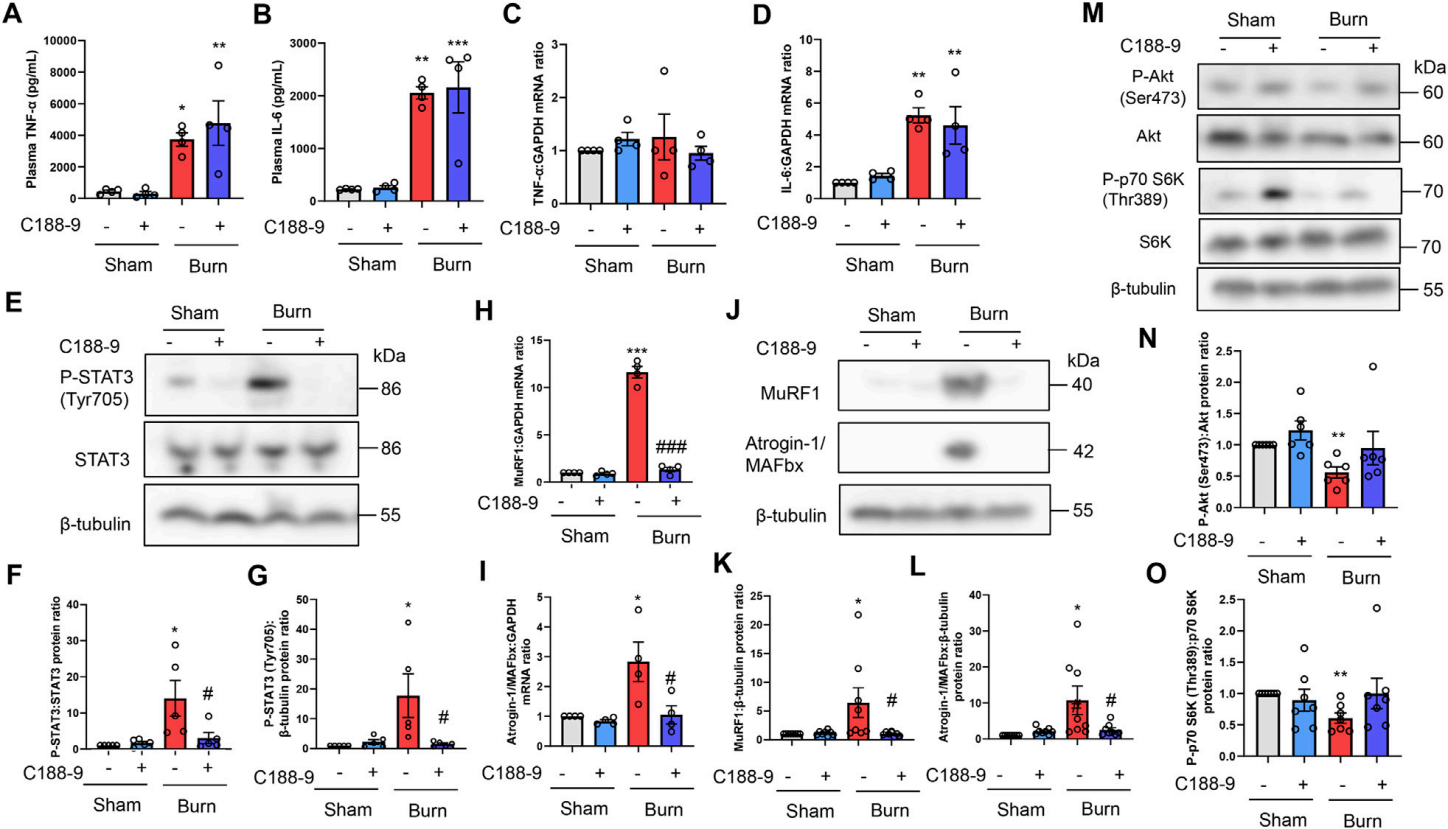
C188-9 reduces burn-induced muscle proteolytic pathway activation in mice. Third-degree or sham burns were administered to wild-type C57BL/6 mice (12–16-week-old male mice) that were then ip injected with vehicle (5% wt/vol dextrose in distilled water containing 5% vol/vol DMSO) or C188-9 (50 mg/kg) 1 h later. The ip injection of C188-9 or vehicle was repeated every 24 h until sacrifice. Then, 24 h after burn or sham-burn injury, plasma samples were prepared and TNF-α **(A)** and IL-6 **(B)** concentrations were measured by ELISA. N = 4/group. qRT-PCR analysis of TNF-α **(C)** and IL-6 **(D)** mRNAs in TA muscles 24 h after burn or sham-burn injury. Data were normalized to GAPDH mRNA levels and are shown as fold increase over the sham-vehicle group. N = 4/group. Western blot analysis **(E)** and quantification of P-STAT3 **(F,G)** in TA muscles 24 h after burn or sham-burn. Data were normalized to STAT3 protein levels or β-tubulin protein levels **(G)** and the ratio in the sham-vehicle group was set as 1. N = 5/group. qRT-PCR analysis of MuRF1 **(H)** and Atrogin-1/MAFbx **(I)** mRNAs in TA muscles 24 h after burn or sham-burn injury. Data were normalized to GAPDH mRNA levels and are shown as fold increase over the sham-vehicle group. N = 4/group. Western blot analysis **(J)** and quantification of MuRF1 **(K)** and Atrogin-1/MAFbx **(L)** expressions in TA muscles 24 h after burn or sham-burn injury. Data were normalized to β-tubulin protein levels, and the ratio in sham-vehicle group was set as 1. N = 8/group. Western blot analysis **(M)** and quantification of P-Akt **(N)** and P-p70 S6K **(O)** expressions in TA muscles 24 h after burn or sham-burn. Data were normalized to Akt and p70 S6K protein levels, respectively, and the ratio in the sham-vehicle group was set as 1. N = 6–7/group. For all panels, data are presented as the mean ± s.e.m.***p < 0.001, **p < 0.01, *p < 0.05 vs. sham injury with vehicle-treated group: ###*p* < 0.001, #*p* < 0.05 vs. thermal burn injury with vehicle-treated group. P-values were derived from one-way ANOVA followed by Tukey’s honest significant difference test or Kruskal-Wallis test followed by Dunn’s *post hoc* tests with Bonferroni correction.

To determine whether the thermal burn-induced skeletal muscle wasting observed *in vivo* were mediated by the STAT3-stimulated activation of proteolytic pathways, we next injected third-degree burn and sham-burn mice ip with vehicle or C188-9 (50 mg/kg) as described in the Methods. Because STAT3 is a downstream effector of IL-6 signaling, the pharmacological inhibition of STAT3 by C188-9 may not reduce the secretion of IL-6 or other inflammatory cytokines in response to thermal burn insults. As expected, treatment with C188-9 did not abolish circulatory levels of TNF-α and IL-6 24 h after third-degree burn injuries (Figures 5A,B). In agreement with the findings in plasma, TNF-α and IL-6 mRNA levels in TA muscles were not reduced in burn mice treated with C188-9 compared with TA muscles from burn mice treated with vehicle alone (Figures 5C, D). Further analysis of the TA muscle extracts revealed that C188-9 significantly reversed the effects of third-degree burns on STAT3 signaling (Figures 5E–G), and its downstream components of MuRF1 and Atrogin-1/MAFbx at the transcriptional (Figures 5H,I) and post-transcriptional levels (Figures 5J–L). The effect of C188-9 on impaired growth signals in TA muscles of burn mice was limited (Figures 5M–O) and it did not alter apoptosis or pro-apoptotic signals (Supplementary Figure S3 in the Supplementary Material) in burn or sham-burn mice. Collectively, these results suggest that the preventive effect of C188-9 on thermal burn-induced skeletal muscle atrophy was associated with the amelioration of proteolytic pathways rather than protein synthesis and apoptosis pathways.

### C188-9 ameliorates C2C12 myotube atrophy induced by plasma from burn mice by reducing STAT3 and proteolytic pathway activation

**FIGURE 6:**
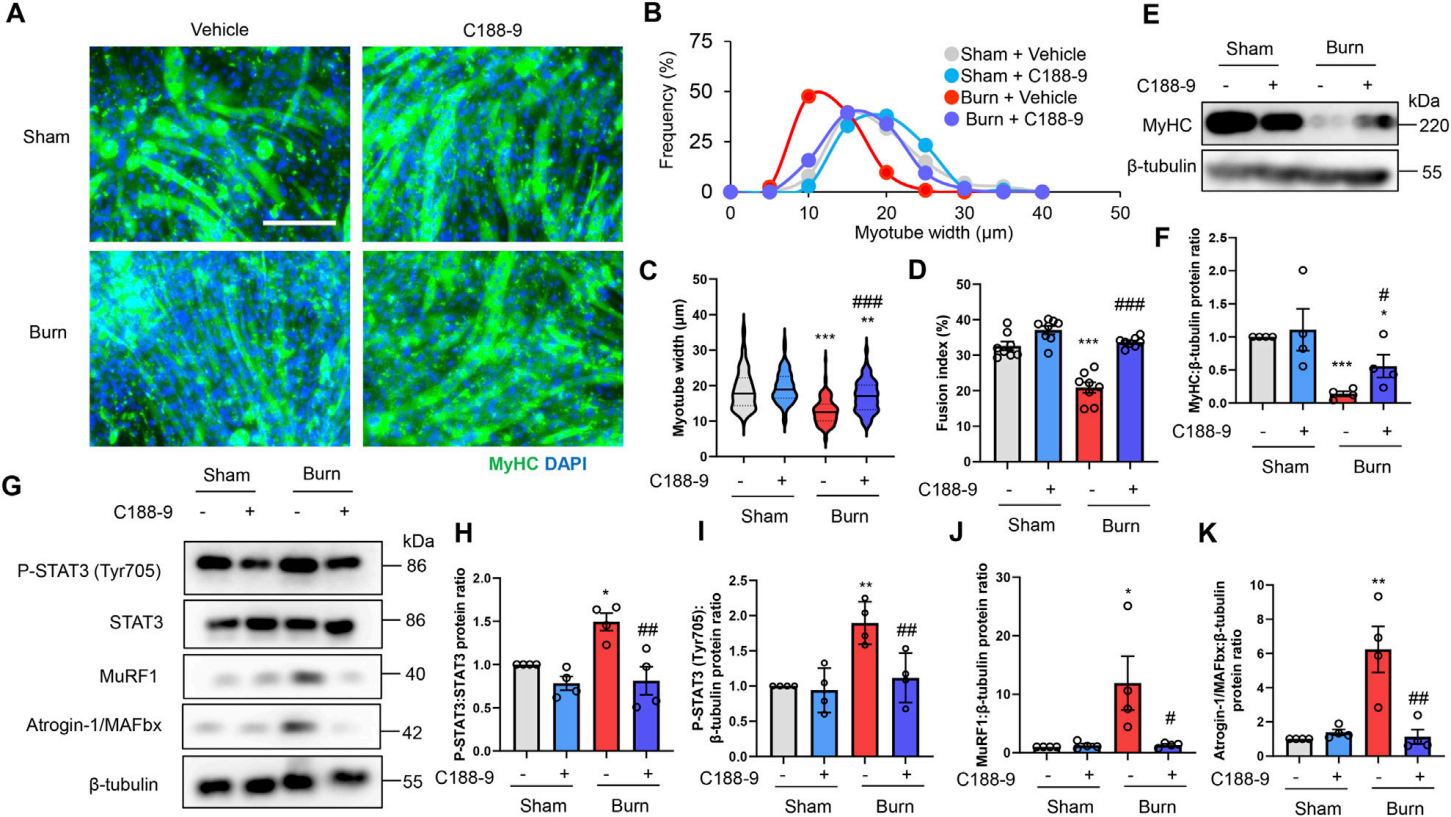
C188-9 reduces proteolytic pathway activation and atrophy of C2C12 myotubes induced by plasma from mice with thermal burn injury. C2C12 myotubes were treated with vehicle (0.1% vol/vol DMSO) or C188-9 (10 µM) and then with plasma collected from mice with third-degree burns or sham-burn injuries (5% vol/vol) 1 h later. Representative immunofluorescence staining of MyHC in C2C12 myotubes **(A)** treated with vehicle or C188-9, and with plasma from burn or sham-burn mice for 48 h. Scale bar = 100 µm. Distribution of myotube widths **(B)**, violin plot of myotube width **(C)**, and fusion index **(D)**. N = 103–127/group. The continuous lines and dotted lines within the violin plot indicate the median and quartiles, respectively. The fusion index was calculated from eight randomly selected fields. Western blot analysis **(E)** and quantification **(F)** of MyHC expression in C2C12 myotubes treated for 48 h with vehicle or C188-9, and with plasma from burn or sham-burn mice. Data in **(F)** were normalized to β-tubulin protein levels, and the ratio in sham-vehicle cells was set as 1. N = 4/group. Western blot analysis **(G)** and quantification of P-STAT3 **(H,I)**, MuRF1 **(J)**, and Atrogin-I/MAFbx **(K)** in C2C12 myotubes treated with vehicle, C188-9, and plasma from burn or sham-burn mice for 24 h. Data were normalized to STAT3 protein levels and β-tubulin protein levels, respectively, and the ratio in sham-vehicle cells was set as 1. N = 4/group. For all panels, data are presented as the mean ± s.e.m. ***p < 0.001, **p < 0.01, *p < 0.05 vs. sham-vehicle cells: ###*p* < 0.001, ##*p* < 0.01, #*p* < 0.05 vs. sham-burn cells. P-values were derived from one-way ANOVA followed by Tukey’s honest significant difference test.

To extend these observations *in vitro*, we treated C2C12 myotubes with DMSO (0.1% vol/vol) or C188-9 (10 µM) and then with plasma collected from mice with third-degree burns or sham-burn injuries (5% vol/vol) 1 h later. These treatment time courses and concentrations of C188-9 were used with reference to previous relevant studies (Bharadwaj, et al., 2016; Huang, et al., 2020). Immunofluorescence staining of myotubes with an MyHC specific antibody demonstrated that exposure to plasma from mice with third-degree burns diminished the diameter and abundance of mature myotubes, which were rescued by C188-9 treatment (Figures 6A–D). Similarly, we observed that MyHC protein expression was also downregulated by plasma from burn mice, but this was reversed by pretreatment with C188-9 (Figures 6E,F). These results indicated that the pharmacological inhibition of STAT3 signaling suppressed muscle protein loss and atrophy in C2C12 myotubes induced by humoral factors related to burn injury. The analysis of cell lysates showed that C188-9 significantly reduced the activation of STAT3 and two ubiquitin ligase proteins, which mediated the effects of plasma from mice with third-degree burn injuries (Figures 6G–K). These results were similar to the responses of burn mice treated *in vivo* with vehicle or C188-9. Taken together, these observations indicate that the pharmacological inhibition of STAT3 prevents humoral factor-mediated skeletal muscle wasting associated with burn injuries by reducing activation of the ubiquitin-proteasomal pathway.

### Schematic summary

**FIGURE 7:**
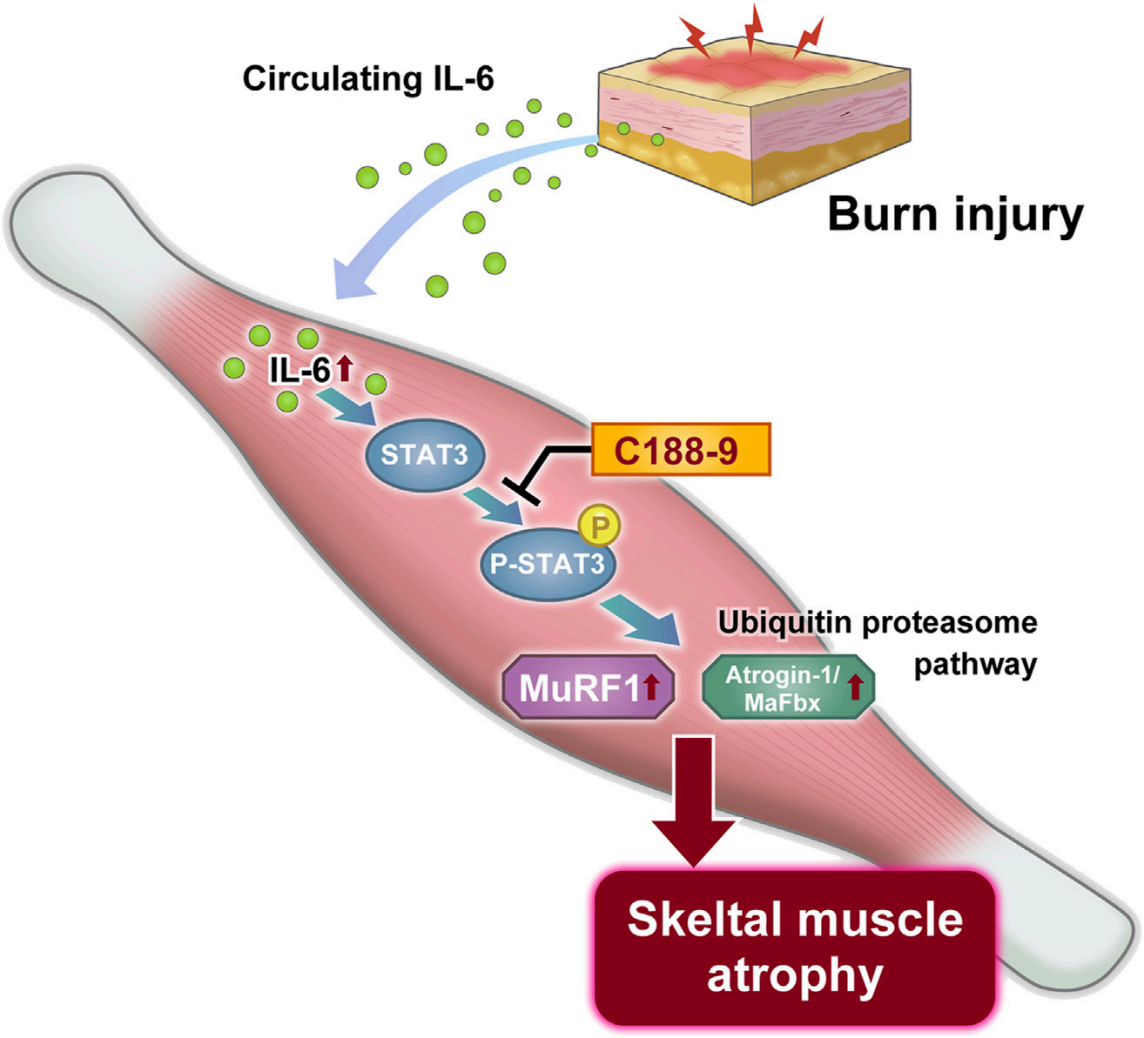
Proposed mechanism of thermal burn injury-induced skeletal muscle wasting.

On the basis of these observations, we propose a mechanistic model for thermal burn injury-induced skeletal muscle atrophy in mice (Figure 7).

## Discussion

Various pro-catabolic conditions, including thermal burn injuries and sepsis, are complicated by progressive skeletal muscle atrophy, which increases the risk of morbidity and mortality in intensive care units (Ali et al., 2008; Hermans et al., 2014; Wang et al., 2020). Currently, the molecular mechanisms that cause muscle loss in thermal burn injuries are not well understood, preventing the development of drugs or other treatment strategies. Our results showed that thermal burn injuries induced systemic inflammation and activated the STAT3 pathway in skeletal muscle by a severity dependent mechanism. When this pathway was activated, there was an increase in the ubiquitin-proteasome system and a decrease in protein anabolic signals that induce skeletal muscle atrophy. The current study also showed that the pharmacological inhibition of the STAT3 signaling pathway by C188-9 reduced the activation of the ubiquitin-proteasome degradation pathway and restored muscle wasting in mice with thermal burn injuries. Pretreatment of C2C12 cells with C188-9 also reduced the activation of the same inflammatory and proteolytic pathways, and reversed myotube atrophy induced by plasma from mice with third-degree burn injuries. Our finding that blockade of the STAT3 signaling pathway ameliorated skeletal muscle atrophy suggests a potential new strategy for clinical interventions in skeletal muscle wasting induced by thermal burn injury.

The presence of a persistent systemic inflammatory response is one of the distinctive features of burn-induced skeletal muscle wasting (Merritt et al., 2012; Shelhamer et al., 2015; Kashiwagi et al., 2017). Several clinical studies found distinct patterns of increased levels of circulating inflammatory cytokines and inflammatory muscle gene expressions in patients with severe burn injuries (Merritt et al., 2012; Merritt et al., 2013). Our finding of increased IL-6 levels in the systemic circulation and TA muscles in a mouse model of thermal burn injury, which was severity dependent, was consistent with these clinical data. Responses to these inflammatory cytokines might be a potential explanation for STAT3 pathway activation and skeletal muscle atrophy in mice with thermal burn injuries. This idea is supported by a previous study that found the injection of IL-6 into rodents activated STAT3 and stimulated muscle proteolysis (Goodman, 1994). In a mouse model of cancer-induced cachexia, muscle wasting was associated with higher levels of circulating IL-6 (White et al., 2012). We observed that exposing C2C12 myotubes to media containing plasma from mice with third-degree burn injuries activated the STAT3 pathway and reduced myotube size and MyHC protein expression. Furthermore, murine recombinant IL-6 treatment mimicked the reaction of C2C12 myotubes treated with plasma from third-degree burn mice. Our results are in line with earlier observations where serum from human burn victims impaired protein synthesis and reduced mature myotubes, leading to myotube atrophy *in vitro* (Corrick et al., 2015; Pin et al., 2019). These results, together with our findings, collectively indicate that humoral factors from burn victims directly activate STAT3 pathways in skeletal muscle, causing burn-induced skeletal muscle wasting. This study extended these observations by demonstrating that the pharmacological inhibition of the STAT3 pathway reversed skeletal muscle atrophy both *in vivo* and *in vitro*.

Increased skeletal muscle catabolism is also a distinctive feature of burn-induced muscle wasting (Pereira C. et al., 2005; Pereira C. T. et al., 2005). The current study found that thermal burn injury activated the IL-6/STAT3 and ubiquitin-proteasome degradation pathway, resulting in skeletal muscle atrophy, which was severity dependent in mice and in C2C12 myotubes. In contrast, the inhibition of STAT3 signaling by C188-9 reduced the activation of the ubiquitin-proteasome system and prevented thermal burn-induced skeletal muscle atrophy in mice and cells. These results collectively suggest that STAT3 signaling was sufficient and necessary for the activation of the ubiquitin-proteasome proteolytic pathways to induce skeletal muscle atrophy in a model of thermal burn injury. These results are consistent with previous studies that used other models, including cancer (Silva et al., 2015) and chronic kidney disease-induced skeletal muscle atrophy (Zhang et al., 2013). Several clinical studies found that human patients with severe burn injuries had robust increases in circulating IL-6, as well as elevated IL-6 gene expression and ubiquitin-proteasome activity in skeletal muscle distant from the burn site (Merritt et al., 2012; Merritt et al., 2013). Previous studies showed that P-STAT3 increased CCAAT/enhancer binding protein δ expression (Zhang et al., 2013), and that there were CCAAT/enhancer binding protein δ binding elements within the MuRF-1 and Atrogin-1/MAFbx promoters (Silva et al., 2015). In line with these observations, we found that STAT3 inhibition by C188-9 reduced mRNA and protein Atrogin-1/MAFbx and MuRF1 expressions in the TA muscles of mice with thermal burn injuries. Thus, pharmacological STAT3 inhibition to reduce muscle catabolism might be a therapeutic approach for thermal burn injury-induced skeletal muscle atrophy. The pharmacological or genetic inhibition of STAT3 was effective for the treatment of skeletal muscle atrophy induced by chronic kidney disease (Zhang et al., 2013), cancer (Silva et al., 2015), and immobilization (Huang, et al., 2020). Our results, together with these previous findings, suggest the usefulness of STAT3 inhibition for the treatment of skeletal muscle wasting in pro-catabolic conditions.

This study also found that apoptosis signals, determined as an increase in cleaved caspase-3, were relatively unchanged 24 h after burn injury, although several previous studies reported the apoptosis signal was strengthened after burn injuries (Yasuhara et al., 2000; Duan et al., 2009; Song et al., 2015; Nakazawa, et al., 2017b; Sehat et al., 2017). The reasons for these discrepancies are likely to be multifactorial: differences in the burn model, animal species, time course, data measurements, or combinations of these factors may have resulted in different findings. For example, using rat models of thermal burn injury, Duan et al. found apoptotic myonuclei in skeletal muscle were maximal 4 days after burn induction (Duan et al., 2009). Their study suggests that apoptosis may be maximal at a later timepoint than in our study. Although C188-9 did not alter the apoptosis signals in mice with burn or sham burn injuries in our study, Silva et al. found that the inhibition of STAT3 activation suppressed caspase-3 leading to the preservation of muscle mass in a cancer cachexia model (Silva et al., 2015). Furthermore, they observed that P-STAT3 stimulated caspase-3 transcription by a mechanism involving STAT3 binding to the caspase-3 promoter, resulting in the increased expression of pro-caspase-3 (Silva et al., 2015). These results collectively suggest that STAT3 inhibition by C188-9 might be an acceptable treatment for burn-induced skeletal muscle atrophy in terms of apoptosis signals.

Impaired skeletal muscle protein anabolism is also an important determinant of skeletal muscle depletion. This study found that the phosphorylation of p70 S6K (Thr389) and Akt (Ser473) were decreased in the TA muscles of mice with burn injuries and C2C12 myotubes incubated with plasma from burn mice. Our findings are in line with previous studies that used similar *in vivo* and *in vitro* models of burn injuries (Lang et al., 2004; Duan et al., 2009; Corrick et al., 2015; Pin et al., 2019). Similarly, impaired cell growth signals and skeletal muscle atrophy were observed in other conditions, including sepsis (Kazi et al., 2011), endotoxemia (Lang et al., 2010; Ono et al., 2020), cancer (Silva et al., 2015), and chronic kidney diseases (Zhang et al., 2013). Previous studies proposed that postburn impaired anabolic status may be ameliorated through the administration of recombinant human growth hormone (Kim et al., 2016), insulin (Gore et al., 2004), insulin-like growth factor I (Herndo et al., 1999), and the use of a synthetic testosterone analog, oxandrolone (Hart et al., 2001). Although C188-9 treatment did not restore the decreased phosphorylation of p70 S6K (Thr389) and Akt (Ser473) in mice and cells induced by burn insults, our results provide a molecular basis for such skeletal muscle anabolism enhancement strategies to treat burn-induced muscle wasting.

We also observed that third-degree burn injury decreased protein anabolic signals in mice, whereas plasma from mice with third-degree burn injuries activated this pathway in C2C12 myotubes. The reasons for these discrepancies are likely to be multifactorial. Recovery from a burn injury is likely to involve the activation of skeletal muscle satellite cells because they are necessary for muscle tissue repair to damage caused by injury. C2C12 cells are an established mouse satellite cell line, and a previous study showed that serum from burn rats activated satellite cells *in vitro* (Wu et al., 2013). Quintana et al. also found satellite cell activation in rat muscles following burn injury *in vivo* (Quintana et al., 2015). A previous study showed that the JAK/STAT3 signaling pathway activation was associated with the promotion of myoblast proliferation and differentiation, as well as catabolic signal activation, leading to muscle cell atrophy (Corrick et al., 2015; Quintana et al., 2015; Belizário et al., 2016; Yousuf et al., 2017). Our results of C2C12 myotube experiments are in agreement with these previous studies. Different reactions of satellite cells and mature skeletal muscle, differences *in vitro* and *in vivo* settings, or a combination of these factors may have resulted in the discrepancies observed in this study.

This study had several limitations. First, our experiments did not examine the effect of C188-9 on the long-term survival of burn mice. Therefore, we cannot predict that preventing skeletal muscle atrophy leads to improved long-term survival in burn victims. In this study, most mice with third-degree burn injuries were lethargic and had lost their appetite for food, resulting in approximately 10%–15% body weight loss 3 days after burn induction. We and our Animal Care and Use Committees considered that conducting a long-term survival study under such conditions was unethical. According to previous reports, an improvement in skeletal muscle atrophy was associated with prolonged survival in mouse models of cachexia induced by cancer (Zhou et al., 2010) and chronic kidney disease (Zhang et al., 2013).

Second, because food and water were not withheld during the experiment, observed changes between mice treated with vehicle or C188-9 can be attributed to differences in food intake or nutritional status. Our experimental protocol included 3 days for monitoring muscle atrophy and weakness, and our Animal Care and Use Committees did not allow fasting for such a long duration. We also acknowledge that other unmeasured factors, such as reduced motion induced by thermal burn injury may have confounded our results. However, we believe our data has potential relevance for clinical settings, because patients with burn injury and systemic inflammation are likely to become lethargic with a loss of appetite and reduced motion.

Despite these limitations, we used *in vivo* and *in vitro* approaches to reveal the effects of a STAT3 signaling-specific inhibitor on the ubiquitin-proteasome degradation pathway and thermal burn-induced muscle wasting. This study provides a molecular basis and rationale to test the effects of STAT3 inhibitors on thermal burn-induced muscle wasting in clinical trials.

Previous studies proposed that STAT3 pathway activation was associated with the development of muscle atrophy in various endogenous diseases including sepsis (Zanders et al., 2022), cancer (Bonetto et al., 2011; Silva et al., 2015; Rupert et al., 2021), chronic obstructive pulmonary disease (Lin et al., 2021), myocardial failure (Janssen et al., 2005), chronic kidney diseases (Zhang et al., 2013), and degenerative muscle disease (Wada et al., 2017; Liang et al., 2018). Therefore, our data might have significance in conditions beyond muscle wasting associated with thermal burn injury. Our data should stimulate further studies to test the effects of pharmacological STAT3 inhibition on skeletal muscle wasting in such pro-catabolic conditions.

## Conclusion

Our experimental results show that C188-9, which is a selective inhibitor of STAT3 signaling, blocked the activation of ubiquitin-proteasome proteolytic pathways, and ameliorated skeletal muscle atrophy and weakness induced by thermal burn injury. These findings may aid the development of new therapeutic interventions to attenuate thermal burn-induced skeletal muscle wasting.

## Materials and methods

### Animal models of thermal burn injuries

All experimental protocols were approved by the Institute of Animal Care and Use Committee at Kobe University on 10 March 2020 (No. P200202) and performed in accordance with relevant guidelines and regulations. Male C57BL/6 mice at 12–16 weeks of age (The Jackson Laboratory Japan, Yokohama, Japan) with a weight range of 24–29 g were quarantined in a quiet room on a 12-h light/dark cycle for at least 1 week before use. Mice were allowed access to a standard rodent diet (CLEA Rodent Diet CE-7; Clea, Osaka, Japan) and water *ad libitum*. Food intake and body weight were measured every 24 h for 72 h. A second-degree (partial-thickness) thermal burn injury or a third-degree (full-thickness) thermal burn injury comprising 20% of the total body surface area was induced as previously described with minor modifications (Abdullahi et al., 2014; Nakazawa et al., 2017a; Nakazawa et al., 2017b; Medina et al., 2018). Briefly, after anesthetization by the ip injection of medetomidine (0.3 mg/kg; Nippon Zenyaku, Koriyama, Japan), midazolam (4 mg/kg; Astellas Pharma, Tokyo, Japan), and butorphanol tartrate (5 mg/kg; Meiji Seika Pharma, Tokyo, Japan) dissolved in sterile normal saline (Otsuka Pharma, Tokyo, Japan), the dorsum of mice were shaved using an electric clipper (Trimmer Model 2100; Daito Electric Machine, Osaka, Japan). The mice were placed on a plastic mold that exposed the dorsum comprising 20% of the total body surface area, and then they were immersed in 60°C water for 20 s to create a second-degree burn, or for 9 s in 90°C water to produce a third-degree burn (Abdullahi et al., 2014; Nakazawa et al., 2017a; Nakazawa et al., 2017b; Medina et al., 2018). The temperature of water was controlled using a thermostatic water bath (EW-100KD; Asone, Osaka, Japan). Sham-burned mice were immersed in 36°C water for 20 s after general anesthesia. To minimize animal suffering and pain, buprenorphine (0.1 mg/kg; Nissin Pharma, Tendo, Japan) was injected into a subcutaneous layer of the abdomen every 8 h for 72 h after burn or sham-burn injury. For fluid resuscitation, 1 ml of 0.9% NaCl (Otsuka Pharma) was injected ip immediately after the burn or sham-burn. For several time courses (4 h, 1 day, and 3 days after burn induction), mice were euthanized and TA muscles were harvested, immediately frozen in liquid nitrogen, and stored at −80°C until use. The same samples are not used between this manuscript and any of our published works.

### Drug treatments

C188-9, a specific inhibitor of STAT3 signaling, was purchased from Selleck Chemicals (Cat. No. S8605, Houston, TX, USA) and dissolved in 5% wt/vol dextrose in distilled water containing 5% vol/vol DMSO (Sigma Aldrich, St. Louis, MO, USA). Mice were injected ip with 50 mg/kg of C188-9 or vehicle alone 1 h after receiving a third-degree burn injury. Sham-burn mice were injected ip with C188-9 or vehicle as corresponding controls. The ip injection of C188-9 or vehicle was repeated every 24 h until sacrifice. C188-9 doses and administration times were determined with reference to previous relevant articles (Lewis et al., 2015; Bharadwaj et al., 2016; Gavino et al., 2016).

### Survival analysis

Mice were treated as described above and post-treatment acute survival was assessed every 8 h for the first 72 h. This time point was chosen for convenience because buprenorphine was injected every 8 h for 72 h after burn or sham-burn injury.

### Histology

Mice were treated as described above. Three days later, mice were euthanized and the TA muscles were removed, fixed with 4% paraformaldehyde (Fujifilm Wako Pure Chemical, Osaka, Japan) containing 0.2% (wt/vol) picric acid (Fujifilm Wako), embedded in paraffin, sectioned with a thickness of 5 μm, and stained with hematoxylin and eosin (H&E) solution (Fujifilm Wako). Sections were examined and images were captured using a light microscope (AX80 Provis; Olympus, Tokyo, Japan) equipped with a camera (DP70, Olympus). The cross-sectional area of myofibers was quantified with ImageJ software version 1.39 (National Institutes of Health, Bethesda, MD, USA) as previously described (Zhang et al., 2011; Ono et al., 2020). Data are expressed using a violin plot.

### Grip strength test

Forelimb grip strength was measured before and 72 h after burn or sham-burn treatment using a grip strength meter (MK-380Si; Muromachi Kikai, Tokyo, Japan) as described previously (Ono et al., 2020; Fujinami et al., 2021). Briefly, mice were allowed to grab a horizontal bar mounted on the gauge using their front paws, and the tail was gently pulled back. The peak tension was recorded automatically by the implanted sensors in a grip strength meter until the mouse released the grip on the bar. Measurements were repeated in triplicate, and the average of the three measurements was computed.

### Plasma cytokine measurements

Burn or sham-burn were administered to mice as described above. After 4 h or 24 h, mice were anesthetized by the ip injection of 0.3 mg/kg medetomidine (Nippon Zenyaku), 4 mg/kg midazolam (Astellas Pharma), and 5 mg/kg butorphanol tartrate (Meiji Seika Pharma) dissolved in normal saline (Otsuka Pharma). Blood was sampled from the inferior vena cava using a 27 G needle and a syringe (Terumo, Tokyo, Japan), collected into EDTA-2Na tubes, which were centrifuged at 800 × g. for 15 min at 4°C. The plasma layer was collected and stored at −80°C until tested. IL-6 and TNF-α concentrations in plasma were measured using mouse IL-6 and TNF-α ELISA kits (Cat No. KE10007 and KE10002, both Proteintech, Rosemont, IL, USA), respectively, following the manufacturer’s protocol. Absorbance at 450 nm was measured using an iMark™ Microplate Absorbance Reader (Bio-Rad).

### Cell culture

The murine C2C12 myoblast cell line was purchased from the RIKEN Cell Bank (Cell No. RCB0987, Tsukuba, Japan). The myoblasts were cultured in growth medium consisting of high-glucose Dulbecco’s modified Eagle’s medium (Fujifilm Wako), 10% (vol/vol) fetal bovine serum (Equitech Bio, Kerrville, TX, USA), 100 U/mL penicillin, and 100 μg/ml streptomycin (Thermo Fisher Scientific, Waltham, MA, USA) at 37°C in a 5% CO_2_ atmosphere. When the cells reached 80% confluence, the culture medium was changed to differentiation medium, consisting of high-glucose Dulbecco’s modified Eagle’s medium, 2% heat-inactivated horse serum (Thermo Fisher Scientific), 100 U/mL penicillin, and 100 μg/ml streptomycin. The differentiation medium was changed every other day for up to 4 days to induce myoblast differentiation. The resulting C2C12 myotubes were then exposed for 24 h or 48 h to plasma collected from mice with burns or sham-burn injuries as described below.

Plasma collected from mice with burns or sham-burn injuries was heat-inactivated by incubating at 56°C for 30 min using a block incubator (BI-516H; Astec, Shime, Japan). The heat-inactivated plasma was added to the differentiation medium at a final concentration of 5% vol/vol. C188-9 (Selleck Chemicals) was dissolved in DMSO and added to cells at a final concentration of 10 µM 1 h before the addition of plasma. Control cells received an equal volume of DMSO at a final concentration of 0.1% vol/vol 1 h prior to the addition of plasma. In the experiment shown in Supplementary Figure S2 in the Supplementary Materials, murine recombinant IL-6 (Cat. No. 216-16-10 µg; Funakoshi, Tokyo, Japan) dissolved in PBS was added to differentiation medium at a final concentration of 100 ng/ml. These treatment time courses and concentrations of plasma, C188-9, and murine recombinant IL-6 were used with reference to previous studies (Pelosi et al., 2014; Corrick et al., 2015; Bharadwaj, et al., 2016; Pin et al., 2019; Huang, et al., 2020).

### RNA isolation and real-time reverse-transcription polymerase chain reaction (qRT-PCR)

Total RNA was extracted from myotubes or muscle tissues using Isogen (Cat. No. 311-02501; Nippon gene, Tokyo, Japan) in accordance with the manufacturer’s protocol. First-strand cDNA was synthesized from total RNA (1 µg) using random hexamer primers (Cat. No. 3801; Takara Bio, Kusatsu, Japan), RNase inhibitors (Cat. No. 10777019; Thermo Fisher Scientific), a deoxynucleotide mix (Cat. No. 639125; Takara Bio), and Moloney murine leukemia virus reverse transcriptase (Cat. No. 28025013; Thermo Fisher Scientific). The cDNA was diluted five-fold with DEPC-treated water (Cat. No. DR115; BioDynamics Laboratory, Tokyo, Japan) and used as a template for qRT-PCR analysis. qRT-PCR was performed using TB Green Premix Ex Taq II (Cat. No. RR820A; Takara Bio) as described previously (Saito et al., 2021), and included an initial denaturing step at 95°C for 30 s; 45 cycles of denaturing at 95°C for 5 s, annealing at 56°C for 10 s, and extension at 72°C for 15 s. Sequences of the specific primers used were as follows: Atrogin-1/MAFbx, Fw: 5′-CAC​ATT​CTC​TCC​TGG​AAG​GGC-3′, Rv: 5′-TTG​ATA​AAG​TCT​TGA​GGG​GAA​AGT​G-3′; MuRF1, Fw: 5′-CAC​GAA​GAC​GAG​AAG​ATC​AAC​ATC-3′, Rv: 5′-AGC​CCC​AAA​CAC​CTT​GCA-3′; TNF-α, Fw: 5′-TAC​TGA​ACT​TCG​GGG​TGA​TTG​GTC​C-3′, Rv: 5′-CAG​CCT​TGT​CCC​TTG​AAG​AGA​ACC-3′; IL-6, Fw: 5′-CCG​GAG​AGG​AGA​CTT​CAC​AG-3′, Rv: 5′-GGA​AAT​TGG​GGT​AGG​AAG​GA-3′; and glyceraldehyde 3-phosphate dehydrogenase (GAPDH), Fw: 5′-ACC​ACA​GTC​CAT​GCC​ATC​AC-3′ and Rv: 5′-CAC​CAC​CCT​GTT​GCT​GTA​GCC-3′. Detected levels of target mRNAs were calculated by the ΔΔCt method and normalized to GAPDH in arbitrary units, using the Thermal Cycler Dice Real Time System II (Takara Bio).

### Western blot analysis

Muscle tissues and C2C12 myotubes were homogenized and lysed, respectively, in RIPA lysis buffer (Thermo Fisher Scientific) supplemented with 1% protease inhibitor cocktail (Thermo Fisher Scientific). For phosphorylated proteins, a 1% protease and phosphatase inhibitor cocktail (Thermo Fisher Scientific) were applied to RIPA lysis buffer instead of a protease inhibitor cocktail. Western blotting was carried out as previously described with minor modifications (Ono and Sakamoto., 2017; Ono et al., 2020). Briefly, equal amounts of protein (10–20 µg) were separated on 10% or 15% polyacrylamide gels and transferred to polyvinylidene difluoride membranes (Immobilon-P; Merck Millipore, Darmstadt, Germany) using an XCell SureLock System (Thermo Fisher Scientific). Membranes were blocked with 5% (wt/vol) non-fat dried milk for 45 min at room temperature and incubated overnight at 4°C with primary antibodies specific for MyHC (mouse monoclonal, Cat. No. 14-6503, Affymetrix, San Diego, CA, USA; 1:500 dilution), Atrogin-1/MAFbx (mouse monoclonal, sc-166806; Santa Cruz Biotechnology, Santa Cruz, CA, USA; 1:50 dilution), MuRF1 (mouse monoclonal, sc-398608; Santa Cruz Biotechnology, 1:50 dilution), or β-tubulin (rabbit polyclonal, ab6046, Abcam, Cambridge, UK; 1:1000 dilution). Other blots were incubated overnight at 4°C with 5% (wt/vol) bovine serum albumin (Sigma Aldrich) and the following primary antibodies from Cell Signaling Technology (Beverly, MA, USA): P-STAT3 (Tyr705) (rabbit monoclonal, #9145; 1:500 dilution), STAT3 (rabbit monoclonal, #4904; 1:1000 dilution), P-p70 S6K (Thr389) (rabbit monoclonal, #9234; 1:500 dilution), p70 S6K (rabbit monoclonal, #2708; 1:500 dilution), P-Akt (Ser473) (rabbit monoclonal, #9271; 1:500 dilution), Akt (rabbit monoclonal, #4685; 1:500 dilution), Bax (rabbit Polyclonal, #2772; 1:500 dilution), Bcl-2 (rabbit monoclonal, #3498; 1:500 dilution), or cleaved caspase-3 (Asp175) (rabbit monoclonal, #9664; 1:500 dilution). The blots were washed in Tris-buffered saline containing Tween^®^ 20 (0.1% vol/vol, Sigma Aldrich) three times every 15 min and probed with the corresponding horseradish peroxidase-conjugated secondary antibody (1:3000 dilution of goat anti-rabbit IgG, Cat. No. 31460, or goat anti-mouse IgG, Cat. No. 62-6520, Thermo Fisher Scientific). Densitometric analysis of protein bands was performed using Clarity Western ECL Substrate (Bio-Rad) and Amersham Imager 600 image analysis software (GE Healthcare Life Sciences, Piscataway, NJ, USA). When the same blot was analyzed with different antibodies, Restore™ Western Blot Stripping Buffer (Thermo Fisher Scientific) was used according to the manufacturer’s protocol. After removing the original primary and secondary antibodies, the blot was washed three times with Tris-buffered saline containing Tween^®^ 20 (0.1% vol/vol, Sigma Aldrich), blocked with 5% (wt/vol) non-fat dried milk, and re-probed with different primary and secondary antibodies as described above.

### Immunohistochemistry

C2C12 cells were disseminated in poly-l-lysine-coated glass-bottomed dishes (Matsunami Glass, Osaka, Japan) and incubated in growth medium until the cells reached 80% confluence. The cells were then transferred to differentiation medium and cultured for 4 days to induce myoblast differentiation. The resulting C2C12 myotubes were then treated with inactivated plasma from burn or sham burn mice (5% vol/vol) and with C188-9 (10 µM) or DMSO (0.1% vol/vol) for 48 h. At the end of the incubation, the cells were fixed with 4% (wt/vol) paraformaldehyde (Fujifilm Wako) and 0.2% (wt/vol) picric acid (Fujifilm Wako), washed in cold PBS three times every 15 min, and incubated in blocking solution containing 2% (wt/vol) bovine serum albumin (Sigma Aldrich) and 5% (vol/vol) normal goat serum (Jackson ImmunoResearch, West Grove, PA, USA). The fixed cells were then incubated overnight at 4°C in blocking solution with anti-MyHC antibody (mouse monoclonal, Cat. No. 14-6503, Affymetrix; 1:500 dilution). After washing with PBS three times every 15 min, the cells were incubated with Alexa Fluor 488-conjugated anti-mouse IgG (Cat. No. A-11029, Thermo Fisher Scientific; 1:400 dilution) for 1 h at room temperature. Finally, after washing with PBS three times every 5 min, the cells were incubated with 4′,6-diamidino-2-phenylindole (DAPI, Cat. No. D523, Dojindo laboratories, Mashiki, Japan 1:1000 dilution) in PBS for 1 h. After rinsing with PBS three times every 5 min, fluorescence images were acquired using a confocal laser scanning microscope (BZ-x700; Keyence, Osaka, Japan). The widths of 97–127 myotubes from at least five random fields were measured at three points using ImageJ software version 1.39 and averaged for each myotube. Data are expressed using a violin plot. The fusion index, which was defined as the number of nuclei in MyHC expressing myotubes divided by the total number of nuclei (Liu et al., 2018), was also used as a morphological parameter of myoblast differentiation.

### Statistical analysis

Data are presented as the means ± standard error (s.e.m.) unless otherwise indicated. Data were analyzed by *t*-test or one-way analysis of variance (ANOVA) with *post hoc* analysis by Tukey’s honest significant difference test as appropriate. Non-normal distributed data were analyzed by the Kruskal-Wallis test. When a significant difference was detected by the Kruskal-Wallis test, Dunn’s *post hoc* tests were applied with Bonferroni correction. Survival curves were estimated for each group using the Kaplan-Meier method and compared statistically using the log-rank test. All analyses were performed using GraphPad Prism 8 (GraphPad Software, San Diego, CA, USA). A p-value < 0.05 was considered statistically significant.

## Data Availability

The original contributions presented in the study are included in the article/Supplementary Material, further inquiries can be directed to the corresponding author.

## Abbreviations

ANOVA, analysis of variance; DMSO, dimethyl sulfoxide; GAPDH, glyceraldehyde 3-phosphate dehydrogenase; H&E, hematoxylin and eosin; IL-6, interleukin-6; ip, intraperitoneal; JAK, Janus kinase; MyHC, myosin heavy chain; PBS, phosphate buffered saline; qRT-PCR, real-time reverse-transcription polymerase chain reaction; S6K, S6 kinase; s.e.m., standard error; STAT3, signal transducer and activator of transcription 3; TA, tibialis anterior; TNF-α, Tumor necrosis factor-α.
