# Supplementary material for: C188-9, a specific inhibitor of STAT3 signaling, prevents thermal burn-induced skeletal muscle wasting in mice
Source: Front Pharmacol. 2022 Dec 16;13:1031906. doi: 10.3389/fphar.2022.1031906 (PMC9800842; doi:10.3389/fphar.2022.1031906)
Supplement: Supplementary file 1 [file DataSheet1.PDF]

Figure S1. Apoptosis signals and pro-apoptotic signals were not altered at 1 day after thermal burn injury

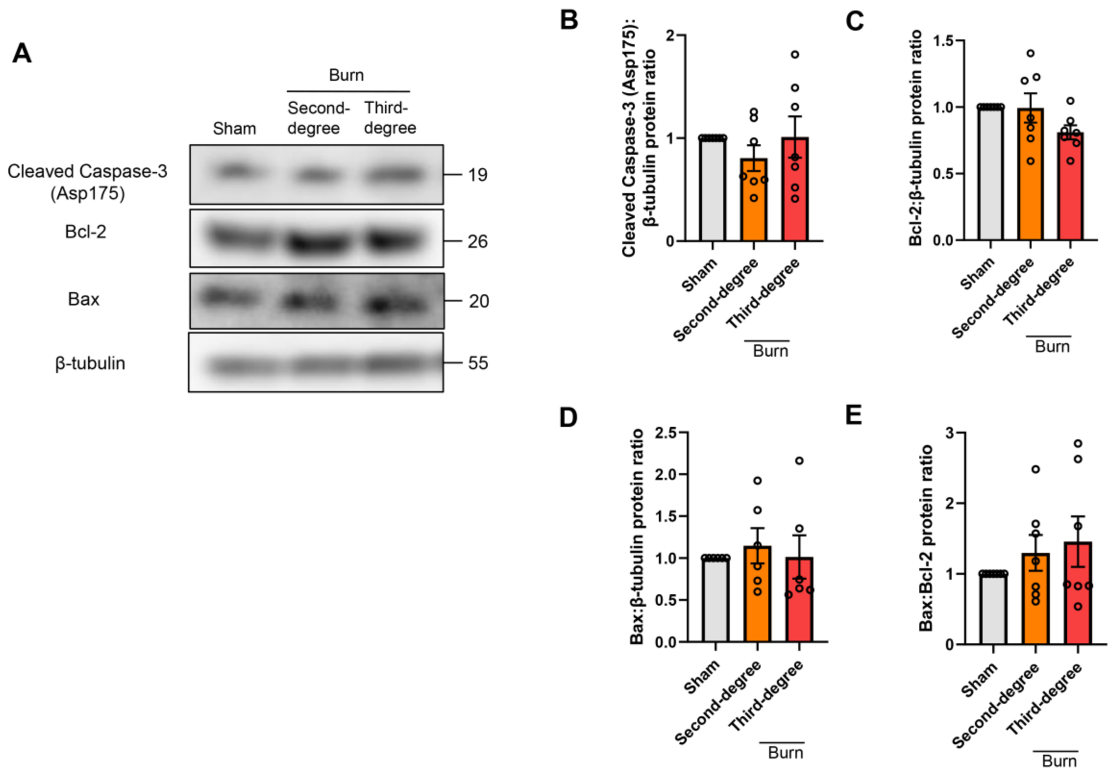

Second-degree, third-degree, or sham burns were administered to wild-type C57BL/6 mice (12-16-week-old males) as described in the Methods. Then, 24 h after burn or sham-burn injury, mice were euthanized, then TA muscles were collected and subjected to western blot analysis. Western blot analysis **(A)** and quantification of cleaved caspase-3 **(B)**, Bcl-2 **(C)**, and Bax **(D)**, and the Bax/Bcl-2 protein ratio **(E)** in TA muscles 24 h after burn or sham-burn. Data were normalized to β-tubulin protein levels and the ratio in sham-control mice was set as 1. N = 7/group.

Figure S2. Exogenous IL-6 activates catabolic and anabolic signals and reduces the expression of MyHC protein in C2C12 myotubes

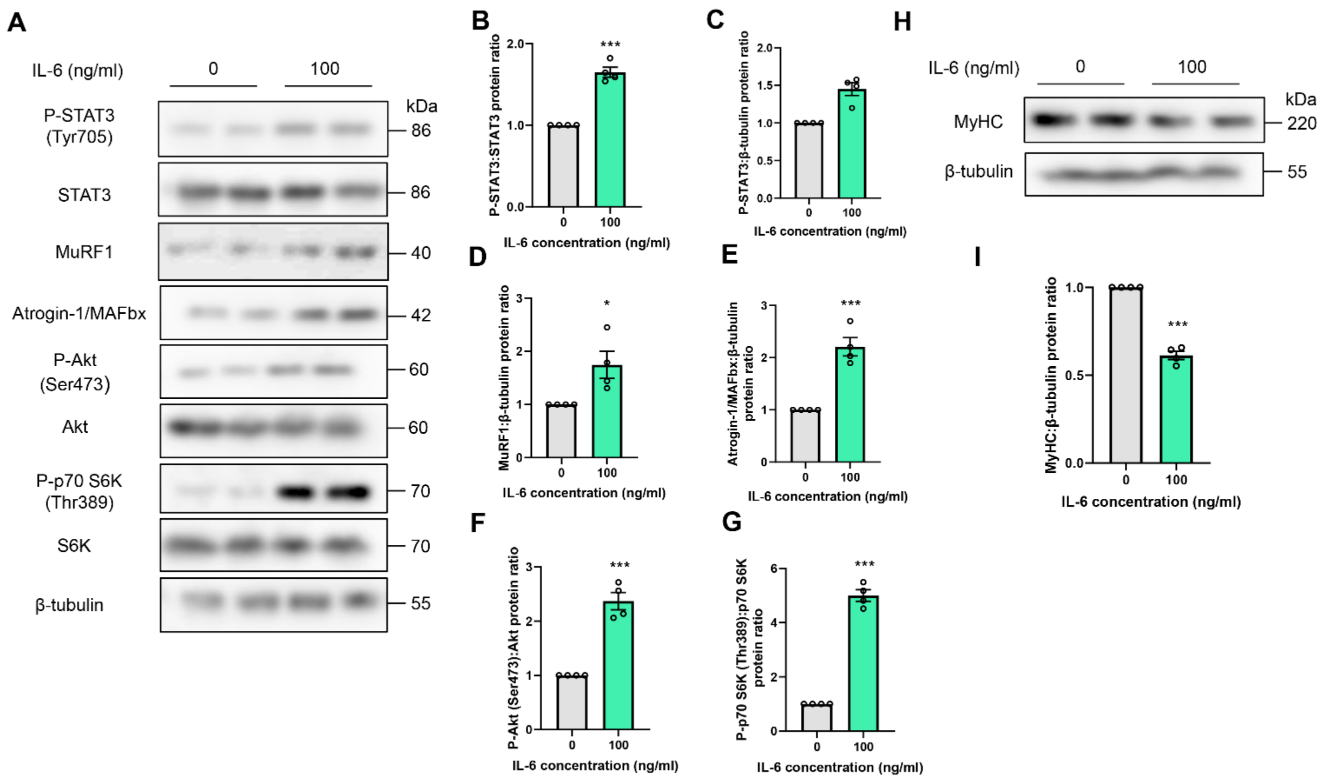

C2C12 myotubes were incubated with murine recombinant IL-6 (100 ng/ml) or vehicle (PBS) for 24 or 48 h as described in the Methods. Western blot analysis **(A)** and quantification of P-STAT3 **(B** and **C)**, MuRF1 **(D)**, Atrogin-I/MAFbx **(E)**, P-Akt **(F),** and P-p70 S6K **(G)** in C2C12 myotubes treated with murine recombinant IL-6 (100 ng/ml) or vehicle for 24 h. Data were normalized to STAT3 protein levels, β-tubulin protein levels, Akt, and p70 S6K protein levels, respectively, and the ratio in vehicle control cells was set as 1. N = 4/group. Western blot analysis **(H)** and quantification of MyHC **(I)** in C2C12 myotubes treated with murine recombinant IL-6 (100 ng/ml) or vehicle for 48 h. Data were normalized to β-tubulin protein levels and the ratio in vehicle control cells was set as 1. N = 4/group. For all panels, data are presented as the mean ± s.e.m. ***p < 0.001, *p < 0.05 vs cells treated with vehicle. P-values were derived from a *t*-test.

Figure S3. C188-9 treatment does not affect apoptosis signals and pro-apoptotic signals at 1 day after thermal burn injury

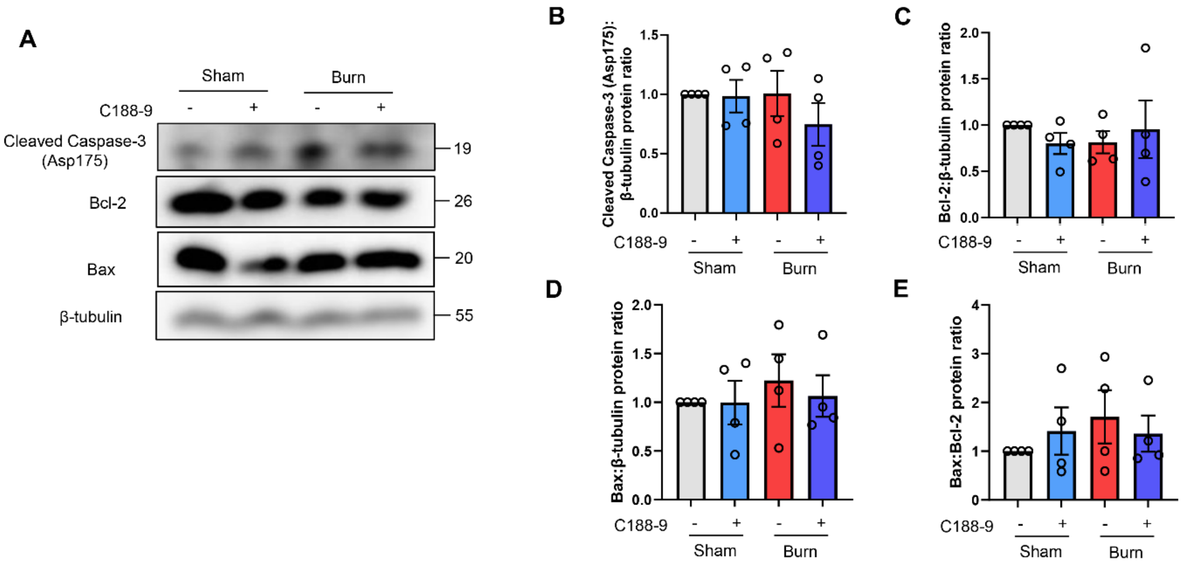

Third-degree or sham burns were administered to wild-type C57BL/6 mice (12–16-week-old male mice), which were then injected ip with vehicle (5% wt/vol dextrose in distilled water containing 5% vol/vol DMSO) or C188-9 (50 mg/kg) 1 h later as described in the Methods. Next, 24 h after burn or sham-burn injury, mice were euthanized, and TA muscles were collected and subjected to western blot analysis. Western blot analysis **(A)** and quantification of cleaved caspase-3 **(B)**, Bcl-2 **(C)**, Bax **(D)**, and the Bax/Bcl-2 protein ratio **(E)** in TA muscles at 24 h after burn or sham-burn. Data were normalized to β-tubulin protein levels, and the ratio in sham-control mice was set as 1. N = 4/group.
